# Supplementary material for: The state of primary healthcare centers in Saudi Arabia: A regional analysis for 2022
Source: PLoS One. 2024 Sep 9;19(9):e0301918. doi: 10.1371/journal.pone.0301918 (PMC11383242; doi:10.1371/journal.pone.0301918)
Supplement: Attachment — Submitted filename: Rresponse Letter.docx [file pone.0301918.s001.docx]

Dear Editor,

I am writing to address the comments and suggestions from Reviewer #2 regarding my manuscript. I appreciate the thorough review and have made the following amendments to enhance the quality and clarity of the paper:

1. **Abstract Corrections**: Spelling and terminology have been corrected, including capitalizing "Primary" and revising "urban" to avoid generalizations about Riyadh and Jeddah.
2. **Results Section**: I have removed the phrase "suggesting regional development efforts" to align the abstract with the results presented.
3. **Spelling and Grammar**: All identified spelling errors, such as the addition of a period after the citation on page 3, line 56, have been corrected.
4. **Terminology**: Alternative phrasing has been used in place of "to cater" and "numerous studies" have been clarified to specify the context within Saudi Arabia.
5. **Flow of Content**: The paragraph on Saudi Arabia's Healthcare Transformation has been revised to improve flow, as suggested.
6. **Section Titles**: The title "Enhancing Healthcare in Alignment with Vision 2030" has been updated to include "Primary".
7. **Structure**: Sections on healthcare enhancement and digital health transformation have been moved to the discussion as recommended.
8. **Acronyms and Definitions**: MOH has been defined at its first mention, and subsequent uses of "Ministry of Health" have been replaced with the abbreviation.
9. **Data Description**: Superfluous descriptions of the data source have been removed, and the term "standardized" has been omitted as suggested.
10. **Table 2 Footnote**: The footnote for Table 2 has been clarified to describe the ranking method accurately.
11. **Paragraph Structure**: Paragraphs on page 6 have been merged for conciseness.
12. **Statistical Data Representation**: The PHCs-per-100,000-people data notation has been standardized to "11.8".
13. **Content Relevance**: Sections recommended for the discussion have been relocated, and the initial descriptive phrase has been eliminated.
14. **Figure Analysis**: The analyses for Figures 1 and 2 have been improved for clarity.
15. **Terminology Consistency**: The terms "urban" and "rural" have been removed to maintain consistency.
16. **Section Expansion**: The section "Impact of Population Growth on PHC Accessibility" has been expanded in depth.
17. **Scope of Paper**: The section "Addressing Non-Communicable Diseases and Health Promotion" has been removed to maintain focus.
18. **Study Description**: The phrase "this comprehensive study" has been refined to "this study".
19. **Conclusion**: Recommendations have been revised to align strictly with the paper's scope.

Each change has been carefully implemented to ensure the manuscript aligns with the journal's standards while maintaining the integrity of the study's findings. I believe the modifications made will address the concerns raised and contribute to the paper's overall rigor.

Thank you for your consideration.

Sincerely,

Waleed M Kattan MD, MHA, PhD

Assistant Professor

Department of Health Services and Hospitals Administration

Faculty of Economics and Administration

King Abdulaziz University, Jeddah, Saudi Arabia

Phone: +966126400000 Ext. 64958

Email: [wmkattan@kau.edu.sa](mailto:wmkattan@kau.edu.sa)
